# Rewiring diversity, physiology, and practice: integrating the next decade of wheat science

**DOI:** 10.1093/jxb/erag143

**Published:** 2026-05-05

**Authors:** Rajeev K Varshney, Reyazul Rouf Mir, Nicola Pecchioni, Matthew Reynolds

**Affiliations:** Centre for Crop and Food Innovation, WA State Agricultural Biotechnology Centre, Murdoch University, Murdoch, WA 6150, Australia; Centre for Crop and Food Innovation, WA State Agricultural Biotechnology Centre, Murdoch University, Murdoch, WA 6150, Australia; Division of Genetics & Plant Breeding, Faculty of Agriculture (FoA), SKUAST-Kashmir, Wadura Campus, Sopore-193201, Kashmir, J&K, India; Research Centre for Cereal and Industrial Crops, Council for Agricultural Research and Economics (CREA), 71122 Foggia, Italy; International Maize and Wheat Improvement Center (CIMMYT), El Batán, Texcoco, Mexico

**Keywords:** Abiotic stress, biotic stress, haplotype-based breeding, heterosis, wheat, wild wheat relatives

**Wheat stands as the backbone of global food security, providing nearly 18% of dietary calories and 19% of protein. The crop faces intensifying climatic extremes, evolving pathogen pressures, resource constraints, and increasing scrutiny regarding environmental sustainability and health narratives. Sustaining genetic gain while broadening resilience and preserving end-use quality represents a defining challenge for contemporary wheat science. Despite these pressures, global wheat production and research have advanced significantly over the past two decades. This Special Issue, based on contributions from the 3rd International Wheat Congress (IWC) 2024 in Perth, Western Australia, presents original research and review articles highlighting emerging themes and advances in wheat research.**

Wheat is the world’s most important cereal crop and serves as a primary staple food for millions of people worldwide (Yao *et al.*, 2025). It constitutes a cornerstone of global agricultural production systems and food security. The wheat yield in two major wheat-producing regions, Asia and Europe, between 2000 and 2020 increased by ∼32% and 25%, reaching average yields of 3.4 t ha^–1^ and 4.1 t ha^–1^, respectively (FAO, 2022). These gains have been supported by remarkable progress in genetics and genomics, advanced breeding methodologies, and the development of improved, climate-resilient wheat varieties around the world. Over the past decade, wheat science has undergone a methodological transformation. Chromosome-scale reference genomes, pan-genomic analyses, and high-density haplotype maps have revealed extensive structural variation and previously underexploited allelic diversity (Jiao *et al.*, 2025; White *et al*., 2025). At the same time, climate change has amplified the frequency of heatwaves and terminal droughts, thus expanding the soil salinity and rising variability in seasonal rainfall patterns, thereby heightening production risks (Anand and Mishra, 2025).

Despite impressive yield gains during the Green Revolution and post-Green Revolution eras, evidence of yield plateaus in several major wheat-growing regions has stimulated renewed discussion in the last years, regarding the limits of conventional selection and the need for new breeding paradigms (Harikrishna *et al.*, 2022). Simultaneous quality-dependent production systems create a persistent sustainability paradox: high grain protein concentrations required for breadmaking quality are strongly linked to nitrogen inputs, yet reducing nitrogen use is essential to lower environmental footprints (Zörb *et al*., 2018; Kang *et al.*, 2026). These intersecting pressures frame a central tension: how to expand genetic diversity and enhance stress resilience while maintaining yield stability, quality, and sustainability. Modern wheat breeding must therefore operate across multiple scales. At the genomic level, breeding strategies must, on the one hand, reintroduce and manage diversity while preserving polyploid stability, and on the other hand learn from gene regulation and sequence diversity to generate new variation by new genomic techniques. At the physiological level, strategies must integrate water relations, carbon allocations, and ion dynamics into predictive frameworks of performance. At the systems level, they must integrate biological and ecological interactions with economics, seed production logistics, nitrogen stewardship, and public perception.

The 3rd IWC, held in September 2024 in Perth, Western Australia and hosted by Murdoch University's Centre for Crop and Food Innovation and the WA State Agricultural Biotechnology Centre, provided a comprehensive platform for leading scientists, researchers, policymakers, and industry stakeholders to share cutting-edge advances in wheat breeding, genetics, genomics, physiology, and agronomy, discuss challenges and opportunities, and foster future research collaborations. This Special Issue explored the full spectrum of emerging themes in wheat research. It includes original research papers and review articles by experts covering a range of topics. This special collection advances a systems view of wheat improvement from wild relative alleles and recombination control to heat imaging, drought hydraulics, salinity reframing, disease resistance, hybrid systems, and societal narratives about wheat (Box 1). Together, these studies chart practical routes towards durable resilience while acknowledging the inherent trade-offs among yield, quality, and sustainability.

Box 1. **Conceptual overview of strategies for improving stress resilience and yield stability in wheat**

Wheat adaptation to major biotic and abiotic stresses, including rust, heat, drought, cold, and salinity, is governed by coordinated morphological, physiological, and molecular responses such as stomatal regulation, osmotic adjustment, membrane stabilization, ion homeostasis (Na^+^/K^+^ balance), ROS detoxification, and protein protection. Expansion of the allelic space through phenotyping-assisted introgression of wild genetic resources helps mitigate domestication bottlenecks and facilitates the incorporation of novel adaptive variation into modern breeding pools. In the context of rust resistance, the integration of haplotype stacking with GWAS-informed trait discovery enables the identification and pyramiding of resistance-associated loci to enhance disease resilience across diverse environments. Coupled with high-throughput phenotyping these approaches strengthen adaptive capacity across genotype × environment × management (G × E × M) interactions by modulating key physiological processes including photosynthetic efficiency, transpiration dynamics, and water-use optimization. Translating trait-level insights into system-level breeding strategies through high-plasticity genotypes, genic male sterility-based hybrid wheat systems, and data-driven decision frameworks facilitates the development of climate-resilient cultivars with improved yield stability, resource-use efficiency, and nutritional value for future wheat production systems. Created in BioRender. Chitikineni, A. (2026) https://BioRender.com/4fpeh0b. AI assistance (Microsoft Copilot) was used to generate some sub-figures, which were subsequently edited and finalized by the authors using BioRender. 
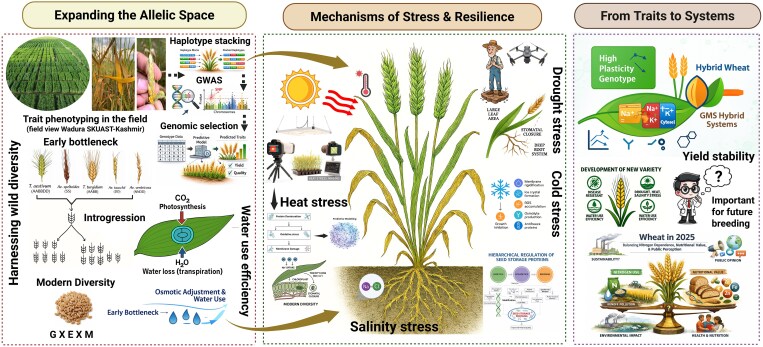

## From genetic diversity to deployable variation: re-architecting the discovery to delivery pipeline

Wheat production and productivity are continuously challenged by a range of biotic and abiotic stresses, further intensified by the growing threats of global climate change (Singh *et al*., 2023). At the same time, the global population is projected to increase by nearly 2 billion people over the next 30 years, placing unprecedented pressure on food systems and sharply increasing demand for wheat (Lam, 2025). Meeting this rising demand is becoming increasingly difficult due to the slowdown in yield gains, driven in part by the progressive narrowing of genetic diversity within modern cultivars (Cheng *et al*., 2024). Centuries of directional selection for yield, adaptation, and agronomic performance have progressively narrowed the genetic base of modern bread wheat, creating a pronounced domestication bottleneck (Cheng *et al.*, 2024). This erosion of diversity has increased the vulnerability of the crop to emerging diseases and intensifying environmental stresses associated with climate change. These limitations, however, can be effectively addressed through the strategic use of wheat wild relatives (WWRs), which harbour extensive untapped genetic diversity for resilience traits such as drought, heat, salinity tolerance, and durable disease resistance. Integrating this wild diversity into breeding pipelines offers a powerful route to restoring genetic breadth and strengthening the adaptive capacity of modern wheat. Therefore, to tackle the challenges, efforts are being made to utilize WWRs as a valuable genetic resource for improving wheat resilience and productivity (Farooq *et al.*, 2026).

The progress in wheat now hinges on converting diversity derived from wild relatives, historical germplasm, and precise genome edits into deployable haplotypes that can be stacked, tracked, and retained in elite backgrounds. WWRs represent not merely reservoirs of useful alleles, but foundational resources for climate-resilient ideotype design. By integrating quantitative trait locus (QTL) mapping, genome-wide association studies (GWAS), next-generation sequencing, genome editing, and structured pre-breeding pipelines, wild variation can be translated into agronomically viable germplasm, including prospects for de novo domestication. This approach moves beyond catalogues of useful alleles, to outlining actionable pathways through structured pre-breeding, de novo domestication, high-resolution mapping, and genome editing, illustrated by the domestication potential of Triticum araraticum as a model for designing future-ready ideotypes (Farooq et al., 2026). Mobilizing this diversity requires careful management of polyploid meiosis. In tetraploid and hexaploid wheat, crossover number and distribution are tightly regulated, with homologous pairing preferred over homoeologous pairing, a process controlled by major genetic loci such as Ph1 (Pairing homoeologous 1) and Ph2 (Pairing homoeologous 2). Manipulating these pathways specifically by suppressing the Ph1 (attributed to TaZIP4-B2) and Ph2 (TaMSH7-3D) loci offers opportunities for introgression from wild relatives but must be balanced against risks to fertility and genomic stability caused by formation of multivalents, highlighting the nuanced potential of recombination engineering (Sourdille et al., 2026).

Building on controlled recombination and targeted introgression from wild relatives, haplotype-based mapping provides a mechanistic framework for deploying rust resistance alleles into elite and landrace germplasm. Genome-wide linkage disequilibrium (LD) analysis defines thousands of LD-based haploblocks, and introducing a haplotype-based introgression fitness index to translate mapping into breeding decisions guides donor selection and simulated crossing strategies. This approach can accelerate the pyramiding of favourable haplotypes, enabling durable resistance while retaining elite genomic backgrounds (Yadav *et al*., 2026).

Historical perspectives further reinforce the dynamic nature of diversity in wheat, demonstrating how breeding trajectories have continually responded to agronomic needs and environmental pressures. Winter adaptation is increasingly recognized as a developmentally plastic trait governed by allelic diversity and copy number variation within the VRN1–VRN2–FT1 regulatory network and its interaction with photoperiod pathways. We are well beyond the traditional binary classifications of spring and winter growth habits; enabling fine-tuning of wheat flowering responses can be another step forward in adaptation to climate change (Hirsz and Dixon, 2026).

Together, these studies illustrate a continuum from wild diversity, to recombination control, to haplotype-based deployment, emphasizing that sustainable gain depends on both expanding and intelligently directing genetic variation.

## Integrative physiology and predictive phenomes: from mechanisms to field-level performance

Genetic diversity provides raw genetic currency; the resilience emerges from coordinated physiological processes measured dynamically and translated into predictive outcome. Multi-year field analyses of elite winter wheat cultivars have identified divergent, yet equally successful physiological routes to yield stability. These analyses emphasize that stability may arise either from sustained post-anthesis assimilate supply linked to stay-green traits, or from efficient pre-anthesis remobilization supporting longer grains, thus illustrating that resilience reflects integrated source–sink dynamics rather than the influence of single traits (Wang *et al*., 2026).

Technological advances now enable such integration at finer resolution through the development of a non-destructive imaging-to-model pipeline including confocal, computed tomography, optical coherence tomography, and live stomatal platforms paired with artificial intelligence and machine learning (AI/ML) to extract predictive anatomical and physiological features under heat stress. By linking structural traits to water transport and carbon assimilation models, these approaches outline a path toward dynamic, high-throughput phenotyping frameworks. This reframes heat tolerance from a post-hoc phenotype into a real-time, predictive phenome, opening the door to in-season selection and data-driven ideotype design (He *et al*., 2026).

Among the various stresses affecting wheat productivity, salinity remains one of the major constraints and is less considered as a genetic improvement objective; only modest progress has been achieved in breeding for salt-tolerant cultivars. It has been noticed that different wheat species, including durum and bread wheat, exhibit varying levels of tolerance to salinity. The effectiveness and limitations of targeting *SOS1* and *HKT1* pathways to enhance salinity tolerance have been explored, but these approaches remain challenged by issues such as ion toxicity, pleiotropic effects, and limited root capacity for Na^+^ storage. Consequently, greater emphasis is needed on tissue-based tolerance mechanisms, alongside a deeper understanding of gene isoforms and underlying physiological processes (Shabala *et al*., 2026).

Under terminal drought conditions, sustained grain yield has been associated with osmotic adjustment, canopy conductance, and water-use efficiency, supported by transcriptomic signatures and cross-validation between controlled lysimeter systems and field trials (Sadeh *et al*., 2026). At a broader scale, yield plasticity quantified across extensive multi-environment datasets demonstrates that plasticity is both adaptive and partially genetically independent of mean yield, challenging the conventional view that a genotype × environment effect represents statistical noise (Giordano *et al*., 2026).

Collectively, these studies signal a shift from descriptive trait evaluation to dynamic, predictive frameworks capable of guiding breeding under increasing climatic uncertainty.

## Translational pathways for sustainable and acceptable wheat: aligning biology, economics, and society

Translational success depends on integrating biological innovation with agronomic, economic, and societal realities. A reassessment of heterosis in wheat, through evaluation of seed production systems and molecular hypotheses underlying hybrid vigour in a polyploid context, underscores that successful hybrid wheat deployment requires a mechanistic understanding of its genetic basis. In polyploid wheat, heterosis probably arises from a combination of dosage balance, network interactions, epigenetic regulation, circadian regulation and organellar crosstalk, highlighting that hybrid wheat represents a species-specific conceptual frontier (Revell *et al*., 2026).

Quality improvement must advance without compromising yield potential. A hierarchical regulatory network for seed storage proteins (SSPs) has mapped 27 genetic loci-rich clusters, where structural genes, regulators, and quality QTLs co-localize. By focusing on regulatory nodes such as transcription factors (bZIP/DOF/NAC/B3/NF-Y), *cis*-elements (the endosperm box), and chromatin modifiers, it becomes possible to fine-tune breadmaking quality with reduced penalty on starch accumulation and yield, thereby strengthening the genotype–end-use connection (Che et al., 2026).

At the food-systems scale, the interconnection between agronomy, human nutrition, and public perception has become increasingly important. Wheat supplies a significant share of global calories and protein yet faces a sustainability tension associated with nitrogen dependence and often misinformed narratives regarding its health impacts. In this direction, the importance of improving nitrogen-use efficiency and maintaining evidence-based communication regarding the role of wheat in sustainable diets has been highlighted (Shewry, 2026).

Additional perspectives further reinforce the importance of putting translation research into practice. Trends in yield plasticity across multi-environment datasets demonstrate measurable economic value under variable seasons, informing risk-aware varietal recommendations (Giordano *et al*., 2026). Together they argue for pipelines where seed systems, quality, nutrient stewardship, and credible communication are design inputs, not afterthoughts.

These contributions emphasize that durable wheat improvement must treat seed systems, quality traits, nutrient stewardship, and public trust as integral design parameters rather than downstream considerations.

## Beyond the Special Issue: future directions

The collective evidence from this Special Issue indicates that the future of wheat improvement will be defined by the transition from incremental trait selection to integrated, pan-omic crop design, uniting physiological insights, genetic diversity, and advanced biotechnologies. Sustained genetic gains under climate volatility, resource limitation, and evolving market expectations will require coordinated progress in mobilizing genetic diversity, dissecting regulatory networks, predictive phenotyping, and next-generation breeding tools.

A central priority is the strategic expansion and deployment of genetic diversity. Wild relatives and underexplored germplasm harbour valuable alleles for stress tolerance, nutritional quality, and adaptive plasticity. Yet harnessing this potential requires recombination-aware breeding strategies, mitigation of linkage drag, and predictive assessment of allele behaviour in elite polyploid backgrounds. *De novo* domestication and targeted editing of wild alleles offer promising routes, although challenges remain in transformation efficiency, genome completeness, dormancy, flowering time control, and harvest index optimization. Conservation, equitable access, and transparent seed systems must therefore accompany technical innovation to ensure sustainable and inclusive deployment.

Hybrid wheat remains a frontier as fascinating as it is complex, with heterosis in polyploid wheat arising from multilayered interactions among heterozygosity, dosage balance, gene networks, and epigenetic regulation. Holistic, species-specific models of heterosis integrated with economically viable hybrid seed production and genomic prediction of combining ability can enhance exploitation of hybrid vigour. Evaluating reproductive and physiological traits, assimilate remobilization, root distribution, and stress-adaptive pathways with phenomics will clarify cultivar-specific adaptation under fluctuating environments.

Pan-genomics and multi-omics integration will underpin next-generation wheat design. Structural variation, gene copy number diversity, and regulatory polymorphisms identified through resequencing and pan-genome initiatives provide a foundation for haplotype-based breeding. However, complex traits such as yield stability, stress tolerance, and grain quality are governed by multilayered regulatory networks. Present and near future research must make best use of AI to integrate genomics with transcriptomics, epigenomics, proteomics, metabolomics, and phenomics, to identify central regulatory nodes controlling coordinated responses.

In parallel, grain quality remains an important concern. Future research must dissect endosperm-specific regulatory motifs, clarify compensatory interactions among glutenin and gliadin fractions, and resolve environmental modulation of SSP expression under nitrogen, temperature, and light variation. Given the competition for substrate and energy between SSP and starch biosynthesis, regulatory-level interventions are needed to decouple yield–quality trade-offs. Evaluating pleiotropic effects of SSP regulators on plant height, phenology, and yield components will guide the development of cultivars tailored to diverse processing, nutritional, and health objectives. Advancing abiotic stress resilience requires shifting from Na^+^ exclusion to tissue tolerance, emphasizing vacuolar sequestration, cytosolic K^+^ retention, isoform-specific channel regulation, and the contributions of the D genome. Knowledge of ion transporter structure–function, voltage and reactive oxygen species (ROS) sensitivity, and integration into signalling networks will refine breeding for ion homeostasis.

Complementary to these advances, gene network modelling and AI/ML frameworks must be refined to handle heterogeneous datasets and imbue interpretability. Explainable AI can help link physiological and anatomical traits with genetic markers, enabling predictive breeding tools that account for genotype × environment interactions. Calibration with large-scale, high-resolution phenotyping data will improve model generalizability across diverse germplasm. Next-generation breeding technologies, including precision editing (CRISPR, base, and prime editing), speed breeding, doubled haploid propagation, and marker-assisted selection, can fine-tune regulatory elements, generate novel alleles with pleiotropic benefits, reduce linkage drag, and accelerate allele pyramiding for yield, quality, and stress resilience. Likewise, epigenetic breeding, leveraging heritable regulatory variation, has potential to modulate gene expression without altering DNA sequence.

Ultimately, accelerating wheat improvement requires convergence across genetics, biotechnologies, predictive phenomics, agronomy, economics, and food systems. Treating seed systems, quality targets, nutrient management, and societal perception as design inputs will advance wheat science towards durable, resilient, and socially responsive crop design suitable for a changing climate and a growing global population.
